# 780 Quality Improvement of the Road Rash Patient

**DOI:** 10.1093/jbcr/irac012.331

**Published:** 2022-03-23

**Authors:** Mariah Sturges, Jason M Bregg

**Affiliations:** AHN West Penn, Pittsburgh, Pennsylvania; West Penn Hospital, Pittsburgh, Pennsylvania

## Abstract

**Introduction:**

What is road rash? “Road rash is a unique burn injury due to imbedded foreign debris and deeply seeded bacteria. Literature on this injury fails to address its unique mechanism and ways to reduce scarring and infection.” (Collier 2020) In the study they found that road rash was not an easy subject to find information on. They did a systematic study to see what information was readily available on road rash. They found that only 24 pertinent articles were found.

Our policies and procedures are based on that of the American Burn Association within the Burn Center. When creating the Road Rash protocol. Use used the same transfer criteria that the American Burn Association identifies. As for the trauma aspect we have covered by ensuring that the trauma is ruled out by a hospital in our network before coming to our center to treat the wound.

**Methods:**

We began to notice that most of the road rash patients that were transferred to us, had not gotten proper care in the outside hospital. The situation would be a patient who should have been directly transferred to us, came to the outpatient clinic where we then would have to perform a very painful dressing change that would have been avoided with the proper methods of transfer and education. We first gathered the data of the transfers that had not gone correctly.

The data that we are looking at for our road rash patients are as follows; TBSA , depth of injury. Location of injury, age, socioeconomic status, type of dressing used, and photos taken before transfer.

We had used data from the year 2019- March of 2021. Total amount was 5 patients. Of these patients 0/5 had proper dressings. correct TBSA, or were transferred properly.

We then gathered our information and spent time hitting our trauma centers within our network. We gave presentations to the emergency department members.

From there we created a list for when we saw the patients in our inpatient unit or our clinic. We wanted to look at the care done before coming to the burn center and what we actually did at the burn center. We looked at TBSA, Location of injury, Age, type of dressing and if there were photos taken

**Results:**

The resulted we wanted to show us if our teaching at these hospitals were effective. Of the 12 patients seen all the patients had the correct dressings on for the time of follow-up, 7/12 had pictures taken for the EMR. All 12 also had the right criteria for transfer or to follow-up in the clinic. The last result of the TBSA was not done correctly on all 12 of the patients.

**Conclusions:**

In conclusion, This project had positive results in the fact that the teach was affective for the dressing changes and taking pre admission photos. We even saw an increase in the number of road rash patients we treated from 2019-2021. The negative results being the improper calculation of TBSA. This showed us where we need to adjust for the next presentations.

